# A QSAR/QSTR Study on the Environmental Health Impact by the Rocket Fuel 1,1-Dimethyl Hydrazine and its Transformation Products

**DOI:** 10.4137/EHI.S889

**Published:** 2008-07-18

**Authors:** Lars Carlsen, Bulat N. Kenessov, Svetlana Ye. Batyrbekova

**Affiliations:** 1 Awareness Center, Hyldeholm 4, Veddelev, DK-4000 Roskilde, Denmark; 2 Center of Physico-Chemical Methods of Investigations and Analysis of al-Farabi Kazakh National University, 95A, Karassai batyr str, Almaty 050012, Kazakhstan

**Keywords:** QSAR, QSTR, rocket fuel, heptyl, hydrazines, transformation products

## Abstract

QSAR/QSTR modelling constitutes an attractive approach to preliminary assessment of the impact on environmental health by a primary pollutant and the suite of transformation products that may be persistent in and toxic to the environment. The present paper studies the impact on environmental health by residuals of the rocket fuel 1,1-dimethyl hydrazine (heptyl) and its transformation products. The transformation products, comprising a variety of nitrogen containing compounds are suggested all to possess a significant migration potential. In all cases the compounds were found being rapidly biodegradable. However, unexpected low microbial activity may cause significant changes. None of the studied compounds appear to be bioaccumulating.

Apart from substances with an intact hydrazine structure or hydrazone structure the transformation products in general display rather low environmental toxicities. Thus, it is concluded that apparently further attention should be given to tri- and tetramethyl hydrazine and 1-formyl 2,2-dimethyl hydrazine as well as to the hydrazones of formaldehyde and acetaldehyde as these five compounds may contribute to the overall environmental toxicity of residual rocket fuel and its transformation products.

## Introduction

Russia, China and Kazakhstan launch rockets from continental space centers. Thus the environment may be more or less affected due to the spread of harmful substances directly or indirectly being a result of the space activities. In a recent paper (Carlsen et al. 2007) we reported on the potential environmental and human health impact of unsymmetrical dimethylhydrazine (heptyl) as a result of the space activities at the Baikonur Cosmodrome, which is an important site for rocket launching with in total more than 2 thousand launches of different rocket-carriers. In recent years the Cosmodrome plays an important role in connection to the International Space Station (ISS). Heavy equipment is transported by rocket-carriers of the type ‘Proton’, the propellant used being the unsymmetrical 1,1-dimethylhydrazine, **1**, also known as “heptyl”.

The fall of the first stages of rocket-carriers is accompanied by the spill of unburned **1** in the amount from 0.6 to 4 tons of which 10–30 kg reach the ground and subsequently spread over the surface (Nauryzbaev et al. 2005).

Industrial enterprises, cities electric power stations, railways, big rivers and channels are located in the vicinity of the fall regions and thus potentially being subject to impact of falling rockets. According to the UNDP more than 7.770.000 km^2^ are, due to their ecological condition referred to as “zones of ecological disaster” (UNDP, 2006).

Recently the potential negative effects of **1** reached the newspaper front pages in connection with the destruction of a US satellite, threatening to fall, ostensibly in order to prevent the environmental spread of contents of unburned fuel. Thus, on Feb. 21st, 2008 Pentagon officials said “they think a Navy missile scored a direct hit on the fuel tank of an errant spy satellite late Wednesday, eliminating a toxic threat to people on Earth” as “A fireball and a vapour cloud seen after the strike appeared to indicate the toxic hydrazine fuel had been destroyed” (Malveaux et al. 2008). It was further stated that “the military was analysing data from the strike to confirm that the tank was hit and that no larger pieces of debris escaped detection” (Malveaux et al. 2008).

The traditional approach to study the effect of primary pollutants in the environment has been to look at the specific compound(s). However, within the environment chemical substances undergo transformation processes eventually leading a more or less complete mineralization depending of the actual degradation potential of the compound. During this transformation process a variety of transformation products may be formed. These compounds, i.e. secondary pollutants may constitute a hazard both to the environment based on their inherent bioaccumulating, persistent and toxic characteristics. However, this potentially “hidden” hazard is often overlooked.

The present paper describes a QSAR/QSTR based assessment of the potential environmental health impact of **1** and its transformations products to the extent these substances have been verified in model studies and/or site specific samples from the fall regions in Kazakhstan. Thus, through this theoretical approach a preliminary assessment of a series of environmentally hazardous substances is obtained and may subsequently form the basis for possible regulatory actions.

## Methods

### Physico-chemical data

In the present study the end-points are generated through QSAR modelling, the EPI Suite being the primary tool (EPA, 2008). The EPI Suite comprises a variety of submodules to estimate, e.g. water solubility (log *S_W_*) calculated by the submodule WSKOW, octanol-water partitioning (log *K_OW_*) calculated by the submodule KOWWIN, vapour pressure (log *VP*) calculated by the submodule MPBPWIN, and Henry’s Law constants (log *HLC*) calculated by the submodule HENRY. Sorption to organic carbon was calculated using the submodule PCKOCWIN. The log *K_OW_* values generated in this way are subsequently used to generate bioconcentration factors (log *BCF*) calculated by the submodule BCF program. Substances exhibiting log *BCF* values of >3.0, but <3.70 are assigned a medium bioconcentration potential whereas substances with log *BCF* > 3.70 were assigned a high bioconcentration potential. (EPA, 1999). Substances with log *BCF* < 3.0 were regarded as non-bioaccumulating.

### Environmental persistence

Through the BioWin module (EPA, 2008) persistence predictions were obtained. The submodule BDP3 provides estimates of a substance’s environmental biodegradation rate by calculating the degradation probabilities. The lower the probability the higher the persistence. Eventually BDP3 returns the biodegradation potential as hours, hours to days, days, days to weeks, weeks, weeks to months and months, respectively, depending on the approximate amount of time needed for a “complete” biodegradation (EPA, 2008; Walker and Carlsen, 2002).

**Table:** 

BDP3	Predicted Half-Lives (days)
Hours	0.17
Hours to Days	1.25
Days	2.33
Days to Weeks	8.67
Weeks	15
Weeks to Months	37.5
Months	60
Recalcitrant	180

Substances with half lives >180 days are assigned high persistence potential, the corresponding BDP3 value being <1.75, whereas substances a half-life in the predominant compartment of ≥60 and ≤180 days are assigned medium persistence potential, the corresponding BDP3 value being >1.75 and <2.0 (Walker and Carlsen, 2002).

The fate in the aquatic media was, in addition to the biodegradation estimated as the potential for volatilisation from water. In the present study volatilisation from rivers (water depth 1m, wind velocity 5 m/s and current velocity 1 m/s) and from lakes (water depth 1m, wind velocity 0.5 m/s and current velocity 0.05 m/s) was calculated using the WVOLW in module in EPI Suite (EPA, 2008).

### Environmental toxicity

Toxicities of the investigated substances have been obtained using the ECOSAR (SRC, 1999) that calculates the toxicity of chemicals discharged into water. Both acute (short-term) toxicities and chronic (long-term or delayed) toxicities are calculated by ECOSAR, the calculations being based on the octanol-water partitioning (log *K_OW_*). ECOSAR can run independently or as an integrated part of the EPI Suite.

ECOSAR return the acute as well as chronic toxicities of the substance under investigation to fish (both fresh and saltwater), water fleas (daphnids), and green algae. In some cases also other effects, e.g. toxicy to earthworms are returned.

The acute toxicities are calculated as LC50 values.

### Detection of transformation products

For experimental verification of the relative amount of the transformation products formed headspace analyses were applied. The obtained data are for now of qualitative nature only. Quantitative experimental data will be presented elsewhere. Thus, withdrawal of the gaseous products from the head space above the soil samples at soil temperature 90 °C and 150 °C was used. The subsequent GC-MS analyses were carried out using a HP-Innowax (30 m × 0.25 mm, 0.25 um film) column for separation, the MS-detection being carried out in total ion mode (m/z range 10–150).

## Results and Discussion

The pollution of the Kazakh territories by **1** originating from space activities at the Baikonur Cosmodrome has been intensively investigated (ISTC, 2004; Carlsen et al. 2007) disclosing 1,1-dimethyl hydrazine (heptyl), **1**, in the soil at more than 1000 sites at fall region where burned-out rocket stages had dropped more than 20 years ago.

It appears the **1** is rather persistent in the soil environment and remains in the environment for a significantly longer time than originally anticipated (Adushkin, 2000). Thus, the activities at the Baikonur Cosmodrome have over the years have resulted in a significant pollution of various sites in Kazakhstan with **1** used as rocket fuel. Compound **1** has been reported toxic and constitutes as risk to human health as well as to the environment.

**Figure 1.**
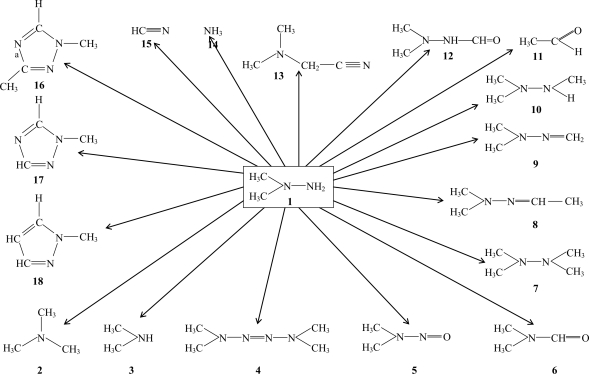
Transformation of 1,1-dimethyl hydrazine in soil and water.

In addition to the pollution with the primary pollutant **1** we have recently been disclosed that a series of so-called secondary pollutants is developed in soil samples polluted by **1**. This group of compounds constitutes both transformation products that are formed directly from **1** as well as compounds that are formed in various consecutive processes. However, a discussion of the actual mechanisms of formation is outside the scope of the present paper and will be described elsewhere. In Fig. 1 the disclosed transformation products are summarized and in Table 1 the basic data of the compounds are displayed.

The compounds **1**–**11** and **13**–**18** have all been detected during our experimental investigations, whereas 1-formyl 2,2-dimethyl hydrazine (12) recently was reported as a transformation product of 1 by (Smolenkov et al. 2007).

Within the new European chemical framework REACH (European Commission, 2006) a special focus is on the persistent, bioaccumulating and toxic (PBT’s) or very persistent and very bioaccumulating (vPvB’s) substances. (European Commission, 2006, Article 57 and 59). These substances are of major environmental concern.

The hazard properties for bulk chemicals are typically linked to the physical-chemical properties such as molecular weight, aqueous solubility, Henry’ Law constant, vapour pressure, and octanol-water partitioning constant and the biodegradation probability (European Commission, 2003).

In Table 2 a series of relevant physico-chemical data for the 18 above-mentioned substances is collected. It is noted that calculated values are in good agreement with the experimentally obtained values available.

It is immediate noted that all compounds exhibit a rather high water solubility, log *S_W_*, and thus a correspondingly low octanol-water partitioning coefficient, log *K_OW_*. In agreement with this we find that these substances display low to very low Henry Law Constants, log *HLC*.

Also the water-organic carbon partitioning, log *K_OC_*, is in all cases low. This strongly indicates that all of these substances potentially possess a significant migration potential and may be transported over significant distances if not chemically or microbially degraded. However, it should be remembered that the majority of the compounds investigated in this study possess acid-base characteristics that may cause a strong affinity to mineral soil particles. Further, although they do exhibit relatively high vapour pressures, log *VP*, they will not easily evaporate from an aqueous phase, whereas the evaporation, if present in the top layers of dry soils may lead to a significant reduction of a possible terrestrial pollution.

Obviously the environmental persistence of a substance is closely related to its probability of degradation as well as its evaporation half life from water. In Table 3 the biodegradation probabilities for the ultimate degradation, BDP3, of the 18 substances are given and transformed into the corresponding time frame together with an assessment of a fast anaerobic biodegradation. Further the calculated residence times in “standard” rivers and lakes (cf. section 2) are collected.

Obviously, all 18 compounds are rapidly degraded the ultimate biodegradation half lives being within weeks apart from N-nitroso dimethylamine (**5**) and dimethylamino acetonitrile (**13**) where the half lives are estimated to be weeks to months. 11 out of the 18 investigated compounds appear to be anaerobically biodegradable whereas the remaining 8 compounds appear not to be (cf. Table 3).

As mentioned above the rocket fuel itself, 1,1-dimethyl hydrazine, **1**, appears to be significantly more persistent than anticipated based on the figures given in Table 3. Thus, Adushkin (2000) found **1** to be very persistent in dry soils suggesting a self-remediation period of lands from **1** of about 34 years. We assume this unexpected high persistence to be a result of minimal microbial activity in the soils investigated possibly as a result of an initial very high concentration of **1** erasing the original microorganisms. Obviously, if an initial high concentration of the primary pollutant, i.e. **1**, prevails erasing the microorganisms this is expected to have a similar effect on the residence times for the secondary pollutants, i.e. compounds **2–18**.

From the data given in Table 3 it is clear that a certain volatilisation from water surfaces and thus moist soils cannot completely be ruled out. Hence neglecting possible biodegradation the evaporation half lives from rivers for the 18 compounds range from a few hours, e.g. compounds **2**, **11**, **15**, to years as for compounds **4** and **12**, respectively. The corresponding evaporation half lives from lakes with significantly lower turbulence are found correspondingly longer ranging from days to years. Applying the PBT criteria previously stated by Carlsen and Walker (2003), i.e. compounds with an aquatic half life >60 days sediment (or terrestrial) half life >180 days being denoted persistent, we conclude that the compounds **1**, **4**, **6**, **10**, **12** and **13** may be regarded as persistent in both rivers and lakes and sediments whereas compounds **7** only in the lake system. Adopting the conservative approach adopting the half life in the lake system as an indication of the sediment half life it is seen that compounds **1**, **4**, **6**, **7**, **10**, **12** and **13** may be denoted as persistent also in the sediment system.

Concerning the bioaccumulation potential for the investigated compounds it has been revealed (cf. Table 2) that in all cases we find log *K_OW_* values <1. Consequently the bioconcentration factors, log *BCF*, were estimated to be 0.5 (default value) (EPA, 2008). For comparison Walker and Carlsen (2002) in their study on persistence and bioaccumulation potential adopted the definition adopted by the USEPA concerning bioconcentration potential (EPA, 1999). Thus, compounds with 1000 < BCF < 5,000 were assigned a medium bioconcentration potential, whereas compounds with BCF > 5,000 were assigned a high bioconcentration potential. It can thus unambiguously be concluded that none of the 18 investigated compounds qualify as being bioaccumulating.

Turning to environmental toxicity the ECO-SAR derived data on the non-polar base line toxicity as well as the polar acute toxicities towards fish, daphnids and green algae have been summarized in Table 4a, whereas the chronic toxicities and the toxicities towards earthworms that have been available in a few cases are collected in Table 4b.

From the figures given in the above Tables (Table 4a, 4b) it becomes immediately clear that the majority of the investigated compounds does not exhibit any significant toxicity towards neither aquatic nor terrestrial organisms. Thus, in relation to acute toxicity significant values are displayed only for the primary pollutant, **1**, and for the compounds **7**–**10**, whereas in the case of chronic toxicities also the 1-formyl 2,2-dimethylhydrazine, **12**, should be taken into account as typically compounds displaying toxicities <1 mg/L should be denoted toxic (Carlsen and Walker, 2003).

In order to evaluate the possible impact on the environmental health by the potential additional toxicity developed through the formation by the transformation products **2**–**17** we need to retrieve some information on the actual amounts of the single compounds generated in soils from originally introduced 1,1-dimethyl hydrazine (**1**).

In a series of experiments we studied the relative amount of compounds **1**–**11**, **12**–**15** and **17**–**18** investigated by sampling and subsequent GC-MS analyses of the gaseous phase in the head space above the soil samples. The data on relative amount of compound **16** was obtained by its extraction from soil using acetone and subsequent GC-MS analysis of the extract. Table 5 displays the data of the analyses. It should be noted that the apparent decrease in the relative concentration observed for some of the compounds with time is associated with the fact that primary formed transformation products may be involved in further transformation processes. The mechanistic studies of these reactions are outside the scope of the present paper and will be reported elsewhere.

The main focus here will be the substances that are both persistent and toxic (*vide supra*). Thus, only 3 compounds, i.e. **7**, **10** and **12**, in addition to the primary pollutant **1** apparently come into play in this context.

Not surprising the compounds **7** and **10**, i.e. tetramethyl- and trimethyl hydrazine, respectively, are found in the group that needs further attention although found in comparable or smaller amounts than **1**. It is seen (Tables 2 and 3) that in any respect studied in the present context compounds **7** and **10** appear to be close to **1**.

It is noted that we did not in our head space analyses confirm the presence of compound **12** among the transformation products as claimed by Smolenkov et al. (2007). However, taking the low vapour pressure and very low Henry’s Law Constant into account (Table 2), both actually being the lowest found among the transformation products studied here, it is not surprising that **12** did not show up among the products found in the head space analyses. Further, compound **12** may display extremely long residence half lives in the river and lakes systems (Table 3) if the microbial activity is low. Hence, based on the persistence and toxicity data it is strongly suggested to include the 1-formyl 2,2-dimethylhydrazine (**12**) among the transformation products that should be further investigated. Thus, it can be concluded that the substances of major concern, including both the primary and secondary pollutants all contain the intact hydrazine structure.

Obviously the two hydrazone structures, i.e. compounds **8** and **9**, apparently should receive some attention as well. They are apparently produced in larger amounts than the hydrazines **7** and **10** (Table 5) but their residence time in the aqueous environment appears to be short (Table 3). However, in the terrestrial environment the lack of microbial activity as discussed above may increase the persistence of these compounds significantly. Thus, based on the toxicity assessment it is suggested that they are further looked for as well. In the cases of the remaining 12 transformation products it appears, solely based on the toxicity assessment that these compounds from an environmental health point of view apparently are of less significance.

## Conclusions and Outlook

The present paper display the use of QSAR/QSTR modelling for a preliminary screening of a series of secondary pollutants arising from a primary pollution with residual heptyl (1,1-dimethyl hydrazine) originating from rocket space activities, the land-based rocket launching at the Baikonur Cosmodrome being used as an illustrative example.

It has been shown that in all cases the transformation products possess a significant migration potential. However, they are apparently rapidly biodegraded within weeks in the aerobic environment. A series of the studied compounds are further found to be anaerobically biodegradable. It should be noted that the primary pollution with 1,1-dimethyl hydrazine may well erase the microbial activity thus prolonging the half lives of the compounds significantly. None of the studied compounds appear to be bioaccumulating.

Substances with an intact hydrazine structure or hydrazones display a toxicity that indicate that transformation products of these types may contribute to the overall environmental toxicity of residual rocket fuel and its transformation products as these compounds display toxicities comparable or even higher than the toxicity of the primary pollutant.

Based on the persistence and toxicity assessments it is concluded that further attention should be given to the transformation products with hydrazine structure, i.e. tri- and tetramethyl hydrazine and 1-fomyl 2,2-dimethyl hydrazine as well as to the hydrazones of formaldehyde and acetaldehyde.

## Figures and Tables

**Table 1.** Structures investigated.

**ID**	**Name**	**CAS No.**	***MW***
1	1,1-Dimethyl hydrazine	57-14-7	60.10
2	Trimethyl amine	75-50-3	59.11
3	Dimethyl amine	124-40-3	45.08
4	1,1,4,4-Tetramethyl tetrazene	6130-87-6	116.17
5	N-Nitroso dimethyl amine	62-75-9	74.08
6	N,N,-Dimethyl formamide	68-12-2	73.10
7	Tetramethyl hydrazine	6415-12-9	88.15
8	Acetaldehylde dimethyl hydrazone	7422-90-4	86.14
9	Formaldehylde dimethyl hydrazone	2035-89-4	72.11
10	Trimethyl hydrazine	1741-01-1	74.13
11	Acetaldehyde	75-07-0	44.05
12	1-Formyl 2,2-dimethyl hydrazine	3298-49-5	88.11
13	Dimethylamino acetonitrile	926-64-7	84.12
14	Ammonia	7664-41-7	17.03
15	Hydrogen cyanide	74-90-8	27.03
16	1,3-Dimethyl-1*H*-1,2,4-triazole	16778-76-0	97.12
17	1-Methyl-1*H*-1,2,4-triazole	6086-21-1	83.09
18	1-Methyl-1*H*-pyrazole	930-36-9	82.11

**Table 2.** Calculated and experimentally determined physico-chemical parameters for the investigated substances^a^.

**No**	**Log *S****_W_***mg/L**	**Log *K****_OW_*	**Log *K****_OC_*	**Log *HLC* atm m^3^ mole^−1^**	**Log *VP* mmHg**
1	1 × 10^6^ (1 × 10^6^)	−1.19	1.29	6.95 × 10^−8^	1.68 × 10^2^ (1.57 × 10^2^)
2	1 × 10^6^ (8.9 × 10^5^)	0.04 (0.16)	1.17	1.28 × 10^−4^ (1.04 × 10^−4^)	1.69 × 10^3^ (1.61 × 10^3^)
3	1 × 10^6^ (1.7 × 10^6^)	−0.17 (−0.38)	1.12	1.81 × 10^−5^ (1.77 × 10^−5^)	1.52 × 10^3^ (1.47 × 10^3^)
4	1 × 10^6^	0.69	1.03	1.96 × 10^8^	21.3
5	9.6 × 10^5^ (1 × 10^6^)	−0.64 (−0.57)	1.58	2.02 × 10^−6^ (1.82 × 10^−6^)	4.3 (2.70)
6	1 × 10^6^ (1 × 10^6^)	−0.93 (−1.01)	0.38	7.38 × 10^−8^ (7.39 × 10^−8^)	3.49 (3.87)
7	1 × 10^6^	−0.52	1.53	7.39 × 10^−7^	1.31 × 10^2^
8	4.5 × 10^5^	0.40	1.85	5.91 × 10^−5^	80.3
9	7.78 × 10^5^	0.68	1.58	4.45 × 10^−5^	3.30 × 10^2^
10	1 × 10^6^	−0.73	1.45	1.53 × 10^−7^	1.45 × 10^2^
11	4.77 × 10^5^ (1 × 10^6^)	−0.17 (−0.34)	0.18	6.78 × 10^−5^ (6.67 × 10^−5^)	9.10 × 10^2^ (9.02 × 10^2^)
12	1 × 10^6^	−1.70	0.65	3.08 × 10^−10^	0.14
13	1 × 10^6^	−0.44	1.00	1.52 × 10^−8^	7.12
14	3.02 × 10^4^ (4.8 × 10^5^)	0.23 (−1.38)	1.16	3.45 × 10^−6^	35.2 (7.51 × 10^3^)
15	3.1 × 10^5^ (1 × 10^6^)	−0.69 (−0.25)	0.43	2.42 × 10^−2^ (1.33 × 10^−4^)	7.32 × 10^2^ (7.42 × 10^2^)
16	2.0 × 10^5^	0.33	2.37	3.60 × 10^−5^	3.78
17	5.7 × 10^5^	−0.21	2.16	3.26 × 10^−5^	10.5
18	7.3 × 10^4^	0.61 (0.23)	1.20	7.88 × 10^−5^	11.5

a Values given in parentheses are experimental values as provided by the database associated to the EPI Suite.

**Table 3.** Calculated persistence of the investigated structures in the environment.

**No**	**BDP3**	**Ultimate biodegradation half life within**	**Fast Aanaerobic biodegradation?**	**Residence half life in rivers^a^**	**Residence half life in lakes^a^**
1	3.0664	Weeks	Yes	272 d	8.1 y
2	2.8137	Weeks	No	5.1 h	5.0 d
3	3.1240	Weeks	Yes	22.9 h	12.8 d
4	2.9425	Weeks	Yes	3.67 y	40.1 y
5	2.6503	Weeks to Months	Yes	11.6 d	130 d
6	2.9834	Weeks	No	282 d	8.4 y
7	3.0044	Weeks	Yes	31.0 d	340 d
8	3.0088	Weeks	No	10.1 h	7.9 d
9	3.0398	Weeks	Yes	12.0 h	8.4 d
10	3.0354	Weeks	Yes	137 d	4.1 y
11	3.1241	Weeks	Yes	6.5 h	5.3 d
12	3.0045	Weeks	Yes	204 y	2220 y
13	2.6761	Weeks to Months	No	4.0 y	44.0 y
14	3.1615	Weeks	No	6.7 d	6.7 d
15	3.1394	Weeks	Yes	2.8 h	3.1 d
16	2.9097	Weeks	No	17.0 h	11.2 d
17	3.0155	Weeks	Yes	17.3 h	11.1 d
18	3.0177	Weeks	No	7.7 h	6.6 d

a h: hours, d: days, y: years. Biodegradation not taken into account.

**Table 4a.** ECOSAR derived baseline and acute toxicity of the investigated compounds (values above 100 are rounded).

**ID**	**LC50 (mg/L)**			**EC50 (mg/L)**

	**Fish^a^**	**Fish^b^**	**Daphnid^c^**	**Green algae**
1	48500	5.9	6.2	0.53^d^
2	4050	290	16.8	16.1^b^
3	4700	300	17.0	15.1^b^
4	2160	1470	1450	830^b^
5	19800	1000	5200	39.8^b^
6	35000	30800	27000	14200^b^
7	18500	4.4	6.1	0.67^d^
8	2850	1.7	3.5	0.53^d^
9	1350	1.1	2.5	0.42^d^
10	23800	4.6	5.8	0.59^d^
11	4600	17.8	48.8	1820^b^
12	200000	14.4	12.2	0.88^d^
13	15100	850	45.5	37.0^b^
14	800	580	550	310^b^
15	8000	6775	6025	3225^b^
16	3700	2675	2550	1450^b^
17	9400	7350	6775	3725^b^
18	1800	1225	1200	690^b^

a Baseline (non polar) toxicity (14 day’s test),

b polar toxicity 96 hrs,

c polar toxicity 48 hrs,

d polar toxicity 144 hrs.

**Table 4b.** ECOSAR derived chronic toxicities and toxicities towards earthworms of the investigated compounds (values above 100 are rounded).

**ID**		**ChV (mg/L)**		**LC50 (mg/kg dw)**

	**Fish**	**Daphnid**	**Green algae**	**Earthworm**
1	0.59	0.62	0.13	–
2	–	–	2.3	–
3	–	–	2.1	–
4	150	–	39.1	1800
5	–	–	4.8	–
6	2475	–	260	3600
7	0.44	0.61	0.17	–
8	0.17	0.35	0.13	–
9	0.11	0.25	0.10	–
10	0.46	0.58	0.15	–
11	8.9	–	52.0	–
12	1.4	1.2	0.22	–
13	–	–	4.7	–
14	56.4	–	11.2	375
15	565	95.2	68.1	1125
16	265	–	55.2	1950
17	665	–	105	2450
18	127	–	31.0	1350

**Table 5.** Formation of transformation products.

**ID**	**Amount detected in headspace**		
	**1 hour**	**1 day**	**1 week**
1	Small	–	–
2	Small	Small	–
3	Small	Very Small	–
4	Large	Medium	Very Small
5	–	–	Very small
6	Small	Small	Small
7	Very small	Small	Very small
8	Small	Medium	Small
9	Very Large	Large	Medium
10	Very small	Small	Very small
11	Small	Small	–
12	No data	No data	No data
13	Medium	Medium	Small
14	Very small	–	–
15	Very small	–	–
16	–	Very small	Small
17	Very small	Small	Small
18	Small	Small	Small
